# Fabrication of high aspect ratio, non-line-of-sight vias in silicon carbide by a two-photon absorption method

**DOI:** 10.1038/s41598-024-52672-6

**Published:** 2024-01-25

**Authors:** Jared E. Payne, Peter Nyholm, Ryan Beazer, Joseph Eddy, Hunter Stevenson, Brad Ferguson, Stephen Schultz, Gregory N. Nielson

**Affiliations:** 1 Department of Electrical and Computer Engineering, Brigham Young University, Provo, UT 84602 USA; 2 Nielson Scientific, Lehi, UT 84043 USA

**Keywords:** Electrical and electronic engineering, Electrochemistry, Ultrafast lasers, Nonlinear optics, Photochemistry

## Abstract

The future of Moore’s Law for high-performance integrated circuits (ICs) is going to be driven more by advanced packaging and three-dimensional (3D) integration than by simply decreasing transistor size. 3D ICs offer low-power consumption, high-performance and a smaller footprint compared to conventional 2D ICs. The key enabling technology to 3D integration is the interposer that provides interconnects to route signals between the chiplets that comprise the IC. However, the fabrication of high-aspect ratio through wafer vias (TWVs), that provide electrical and mechanical connection between chiplets on the top and bottom of the interposer, is one of the important challenges that limit interposer performance. Current fabrication technologies are limited by tapering effects and the need for direct line of sight to the fabrication surface. These limit the possible aspect ratios of vias and require large, complicated surface traces to connect the vias to the chiplets. Here, we demonstrate the fabrication of high-aspect ratio, non-line-of-sight TWVs in silicon carbide (SiC). SiC provides better mechanical, chemical, and thermal performance than silicon (Si). The technique uses an electro-chemical etch process that utilizes two-photon absorption to create any arbitrary 3D structure in SiC allowing for direct, subsurface routing between chiplets.

## Introduction

For the last several decades, Moore’s Law has dictated a reduction in transistor size resulting in a decrease in cost per transistor and an increase in performance. However, scaling of the very large scale integrated (VLSI) circuits is reducing gate delays but increasing interconnect delays. Interconnect loading also affects power consumption in high-performance chips. Due to these challenges and practical and physical limitations on further transistor size reductions, the integrated circuit (IC) industry is turning to the intimate integration of multiple smaller sub-system integrated circuits (i.e., chiplets) within an advanced package, referred to as 3D integration, to continue to deliver the advanced IC performance increases dictated by Moore’s Law^1–4^. Furthermore, there is an increasing need for the integration of different signals (digital, analog, RF) and technologies (SOI, SiGe, HBT, GaAs, SiC, GaN) which is further driving the growth of 3D integration in the IC industry.

3D integration involves using an interposer chip with electrically conductive through wafer vias (TWVs) that integrates two or more chiplets^5–12^. These chiplets can be connected on the same side and/or different sides of the interposer. The TWVs provide the electrical interconnects between the chiplets. For high performance, a high density of interconnects between the chiplets is required. The limit to the density of TWVs in an interposer is determined by the maximum aspect ratio of the TWV, which is defined as length of the via over the diameter of the via.

There are several methods that researchers have used to create TWVs including laser ablation^13–15^, wet etching^16,17^, and deep reactive ion etching (DRIE)^18–25^. DRIE is the most common technique used with 3D integration and provides a maximum aspect ratio of 10–30. The aspect ratios of vias created using this technique are limited due to a tapering effect that occurs as the opening of the holes tend to increase with etch depth^19^. Another limitation that all techniques have is that they require direct line-of-sight to the etching surface. This means that any routing must be done on the surface of the interposer instead of within the substrate’s bulk.

Many high aspect ratio via studies have used Si as their TWV substrate; however, due to the large amounts of heat generated by the connected chiplets, an interposer with a high thermal conductivity is desirable for high performance. Single-crystal silicon carbide (SiC) is a semiconductor that has a high thermal conductivity, an attribute that provides high heat dissipation^26^. There have been a few studies on creating TWVs in SiC, but the methods used (including DRIE) have not been able to create vias with aspect ratios over 17:1^22–24^.

This paper presents a technique to increase the aspect ratio of vias through SiC wafers using a new photo-electro-chemical etching method that takes advantage of a non-linear optical effect in the SiC. In this etch technique, the width of the TWV is not dependent on its depth. Thus, the aspect ratio of TWVs created with this technique can be very high, greater than 100:1. Even more significant is that the demonstrated technique enables the fabrication of non-line-of-sight vias.

**Figure 1:**
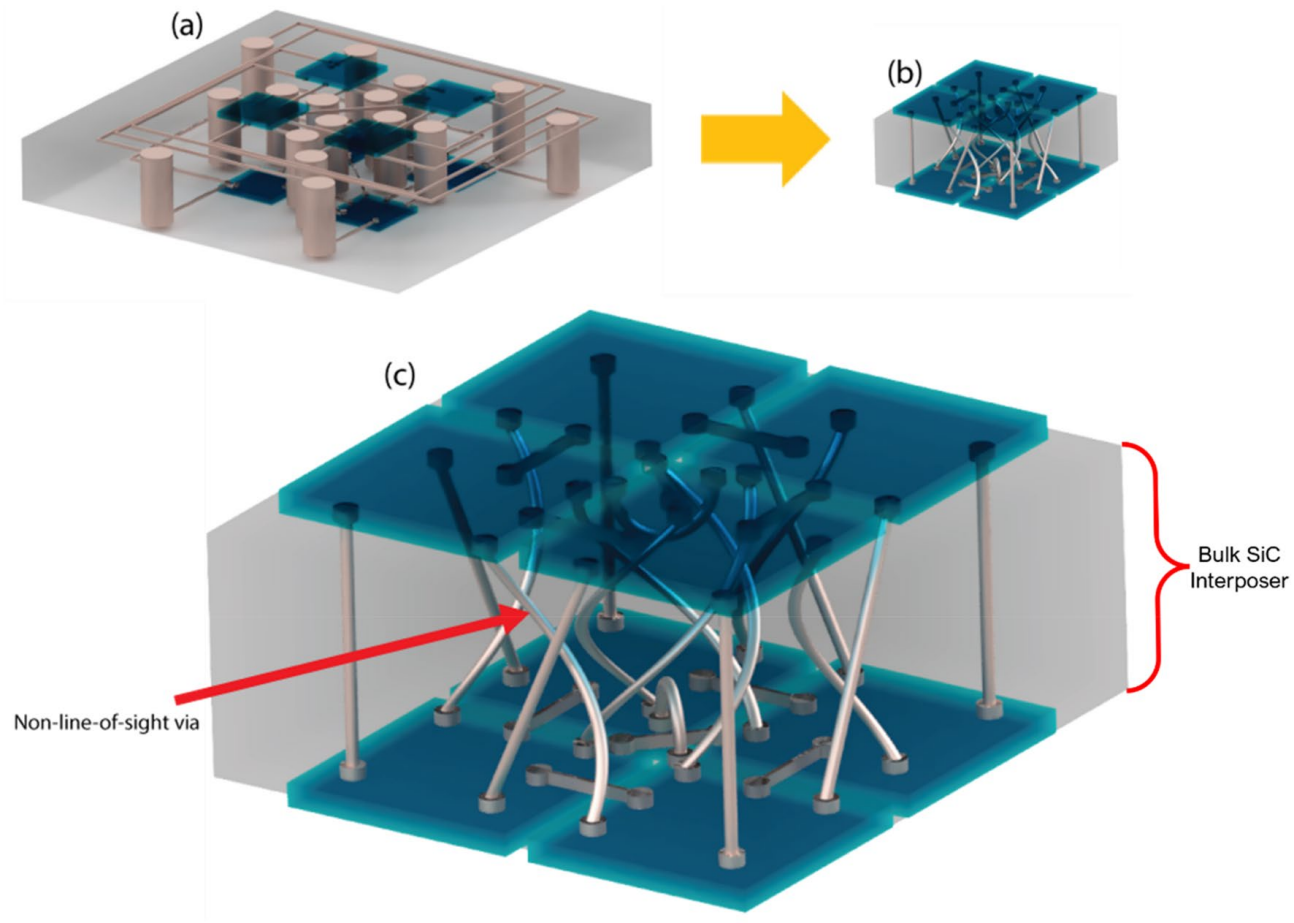
Illustration of the benefit of high aspect ratio, non-line-of-sight through wafer vias in interposers for 3D integration. The eight thin blue elements represent chiplets being interconnect by the interposer. In this illustration each of the chiplets has one connection to each of the other chiplets. In (**a**), low aspect ratio straight vias are used with a single metal layer redistribution layer to provide the interconnects, resulting in a large area interposer with long interconnect lengths. In (**b**) high aspect ratio, non-line-of-sight vias enable very dense interconnects with short interconnect lengths. (**c**) provides a close-up view of the non-line-of-sight vias in (**b**).

Figure 1 is a simple illustration of the benefit of high aspect ratio, non-line-of-sight TWVs for 3D integration. In this illustration, eight chiplets are interconnected in a circuit using an interposer where each chiplet has one direct electrical connection with all other chiplets. Figure 1a shows the large footprint that results from a single layer metal redistribution layer on each side of the interposer combined with TWVs with a low aspect ratio. The requirement for electrical isolation between traces causes the single metal layer to spread out significantly. The TWVs spread the footprint out even further. Figure 1b shows that interweaved, non-line-of-sight and high aspect ratio TWVs results in an increase in the chip density. The result is a decrease in interconnect length at the cost of higher DC resistance. The interconnect latency is given by t = RC = (ρ ε)*L^2^/(WT), where R = ρL/W^2^, C = ε(WL/T), ρ is the resistivity of the metal, ε is the permittivity of the dielectric, L is the via length, W is the via diameter, and T is the via separation. Since the latency is proportional to the length squared the higher aspect ratio vias results in a decrease in the interconnect latency even with an increase in resistance^26^. Furthermore, the TWVs enabled by the 3D etching capability allows for the most direct connection paths between the chips, resulting in an increase in speed, decrease in power consumption, and decrease in the required thermal dissipation. Furthermore, creating high aspect ratio vias also has the potential for the formation of optical waveguide vias.

The innovation is a result of combining electrochemical etching of semiconductor materials with two-photon absorption. The process of electrochemical etching of SiC requires positive charge carriers referred to as “holes” (i.e., electron vacancies in the atomic crystal lattice of the semiconductor). To drive the electrochemical etch process, holes are typically added by either doping the semiconductor or illuminating the semiconductor with light that has a photon energy above the bandgap energy of the semiconductor^27–32^. Our innovation is to introduce holes within the SiC in very precisely controlled locations by focusing sub-bandgap light into the SiC in such a way that the intensity of the focal spot creates conditions necessary for two-photon absorption at that location (and only that location). By controlling where the holes are created, we also control where electrochemical etching occurs. By moving the focal spot of the light within the SiC in x, y, and z dimensions, we can directly create 3D vias and other 3D structures within the SiC without line-of-sight access. The 3D photo-electro-chemical etch technique can be applied to other semiconductors in addition to SiC.

**Figure 2:**
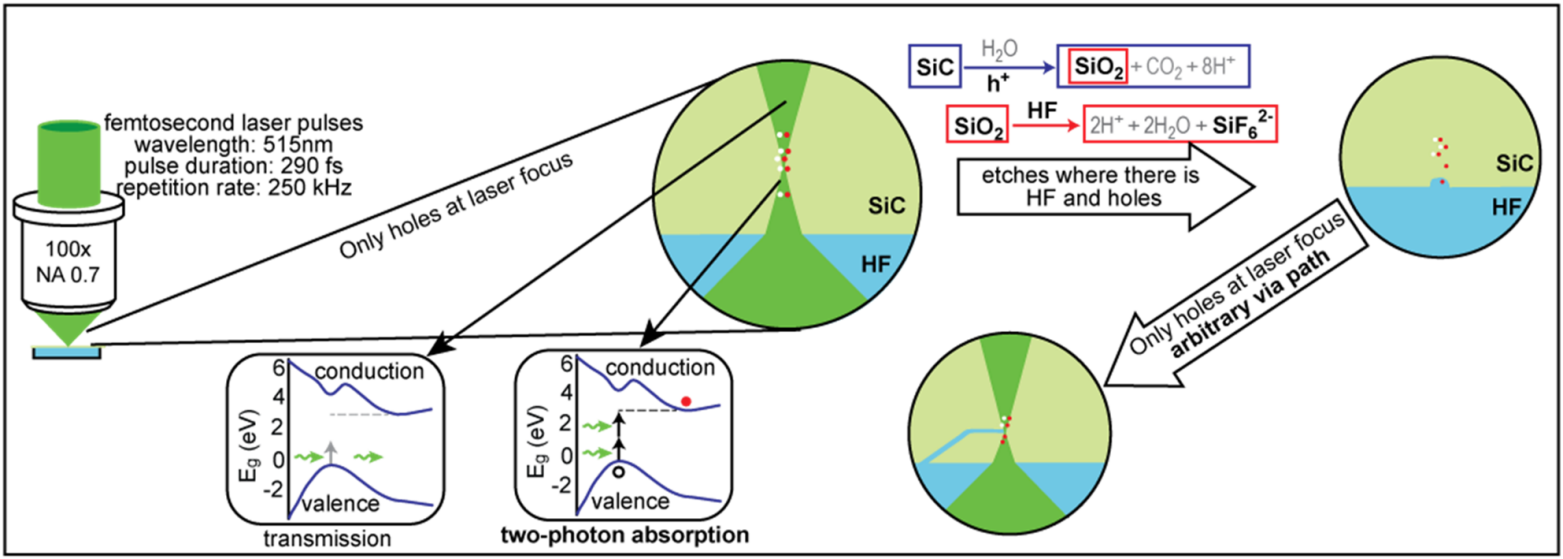
Illustration of the combined two-photon absorption, photoelectrochemical etch process. The femtosecond laser is focused to a small spot within the wafer, which creates holes only at the laser focus through two photon absorption. The electrochemical reaction etches the SiC when there are holes and HF acid solution present, resulting in the creation of non-line-of-sight high aspect ratio vias or other microscale 3D structures.

Figure 2 shows the photo-electro-chemical etching process combining two photon absorption and electrochemical etching. The key to the process is that a femtosecond laser is focused to a small spot within the wafer. The photon energy of the laser is below the bandgap energy of the SiC wafer resulting in the wafer being transparent to the laser. However, at the focus of the laser the energy density is high enough to produce a nonlinear effect where the combined energy of two photons is greater the bandgap of the material resulting in the absorption of the photons and creation of an electron–hole pair. Figure 2 illustrates that the positively charged holes are only generated at the focus of the laser.

The two most common SiC crystal structures 4H-SiC, and 6H-SiC have bandgaps respectively of 3.26 eV, and 3.02 eV^27^. In this demonstration we used 4H-SiC. For this material, ultraviolet light with a wavelength less than 380 nm is absorbed and produces holes directly with linear absorption, while light with a wavelength greater than about 380 nm transmits through the wafer. This demonstration used a femtosecond laser with a wavelength of 515 nm.

At room temperature SiC is a highly chemically resistant material^27^; however, wet etching of SiC has been demonstrated using an electrochemical process^28–33^. The process consists of oxidation of the SiC surface with subsequent oxide etching by hydrofluoric (HF) acid. For silicon dioxide to form, electron holes must be present in the SiC as given by^28^

1 $${\text{SiC }} + {\text{ 4H}}_{{2}} {\text{O }} + {\text{ 8h}}^{{+}} \to {\text{ SiO}}_{{2 }} + {\text{ CO}}_{{2 }} + {\text{ 8H}}^{{+}} ,$$

where h + are holes. Removal of the oxide is then carried out by a reaction with HF as given by

2 $${\text{2SiO}}_{{2}} + {\text{ 6HF}} \to {\text{2H}}^{+} \, + {\text{ SiF}}_{{6}}^{{{2} - }} + {\text{ 2H}}_{{2}} {\text{O}}.$$

Thus, at the focus of the laser the light is absorbed, and holes are produced. Etching occurs at the location of the focal point and only at that location because HF acid and holes are both present. After the SiC around the focal point etches away, the etching stops because the focus of the laser is no longer located within the SiC and thus there is no longer any two-photon absorption and no holes. If the focus of the laser is moved too far away from the interface between the SiC and HF acid, the etching stops because there is no overlap between the generated holes and the HF acid.

Figure 2 illustrates that the novel technique enables the creation of arbitrary 3D vias and other structures by moving the focus of the laser. The via tube is created by placing the acid at the back surface, focusing the laser on the back surface, and then moving the focal point to create an arbitrary 3D structure. The diameter of the via is not dependent on its length. In addition, since the SiC substrate is transparent to the laser, the focus can be placed anywhere. The result is that the via does not require line-of-sight to the surface. Thus, vias can be fabricated directly between the back and front surfaces, or the vias can be routed within the SiC substrate.

## Results

Because of the flexibility of the technique, the possible shapes, sizes, and density of structures are vast. This section illustrates several simple structures created in SiC that highlight the non-line-of-sight, high aspect ratio, and high-density via capabilities.

**Figure 3:**
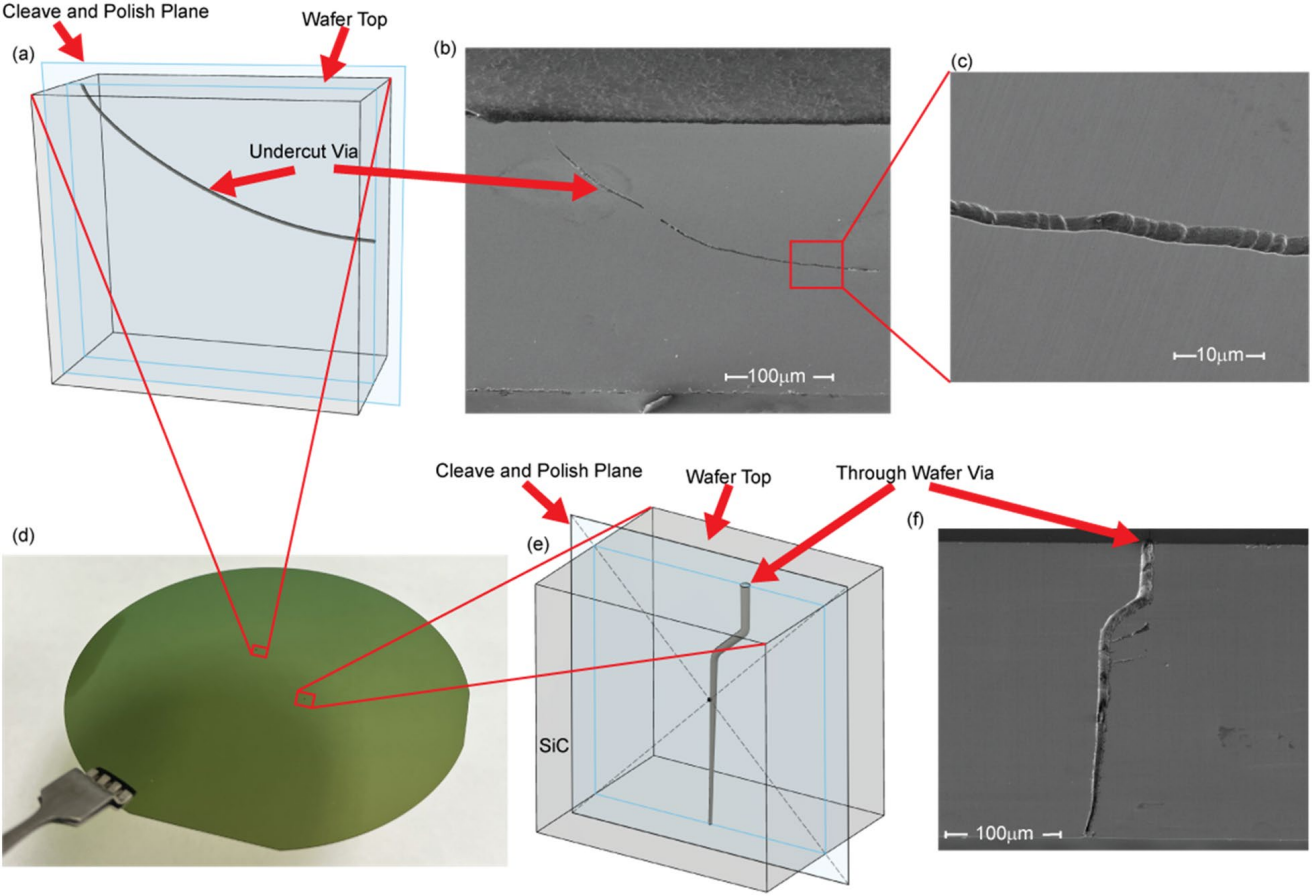
Cross-section, side profile scanning electron microscope (SEM) images of high aspect ratio, non-line-of-sight structures in a single-crystal 4H n-type SiC wafer created using the two-photon photoelectrochemical etch process. (**a**) 3D illustration and (**b**) SEM images of a via microchannel that changes direction to be nearly parallel with the wafer surface, accomplished by controlling the position of the laser focus within the wafer. (**c**) A close-up SEM image of a section of (**b**). The microchannel illustrated in (**a**), (**b**), and (**c**) is 350 µm long and has a consistent 2–4 µm diameter throughout its entire length. (**d**) A photograph of a 100 mm n-type 4H SiC wafer. (**e**) 3D illustration and (**f**) SEM image of a via microchannel that runs completely through a 350 µm wafer. This via microchannel is initially perpendicular to the wafer surface then changes to a 45° angle then resumes a perpendicular orientation. This via microchannel is tapered from a diameter of about 10 µm down to a smaller diameter of 2.5 µm.

Figure 3 shows images of two cross-sections of non-line-of-sight vias fabricated in 4H-SiC. The process to obtain these pictures involves cleaving the SiC near the etch location and polishing the cleaved edge to reveal the via using a high-precision edge polisher. Figure 3 shows the side profile of a long via that begins perpendicular to the wafer surface and then slowly changes direction to run nearly parallel to the wafer surface.

Figure 3d,e show the side profile a non-line-of-sight through-wafer via. Due to the difficulty of the cleave and polish process, this via is intentionally wider to make it easier to polish back and reveal the etch. We have been able to reproduce these non-line-of-sight results several times across different locations in the same wafer and across other wafers as well.

**Figure 4:**
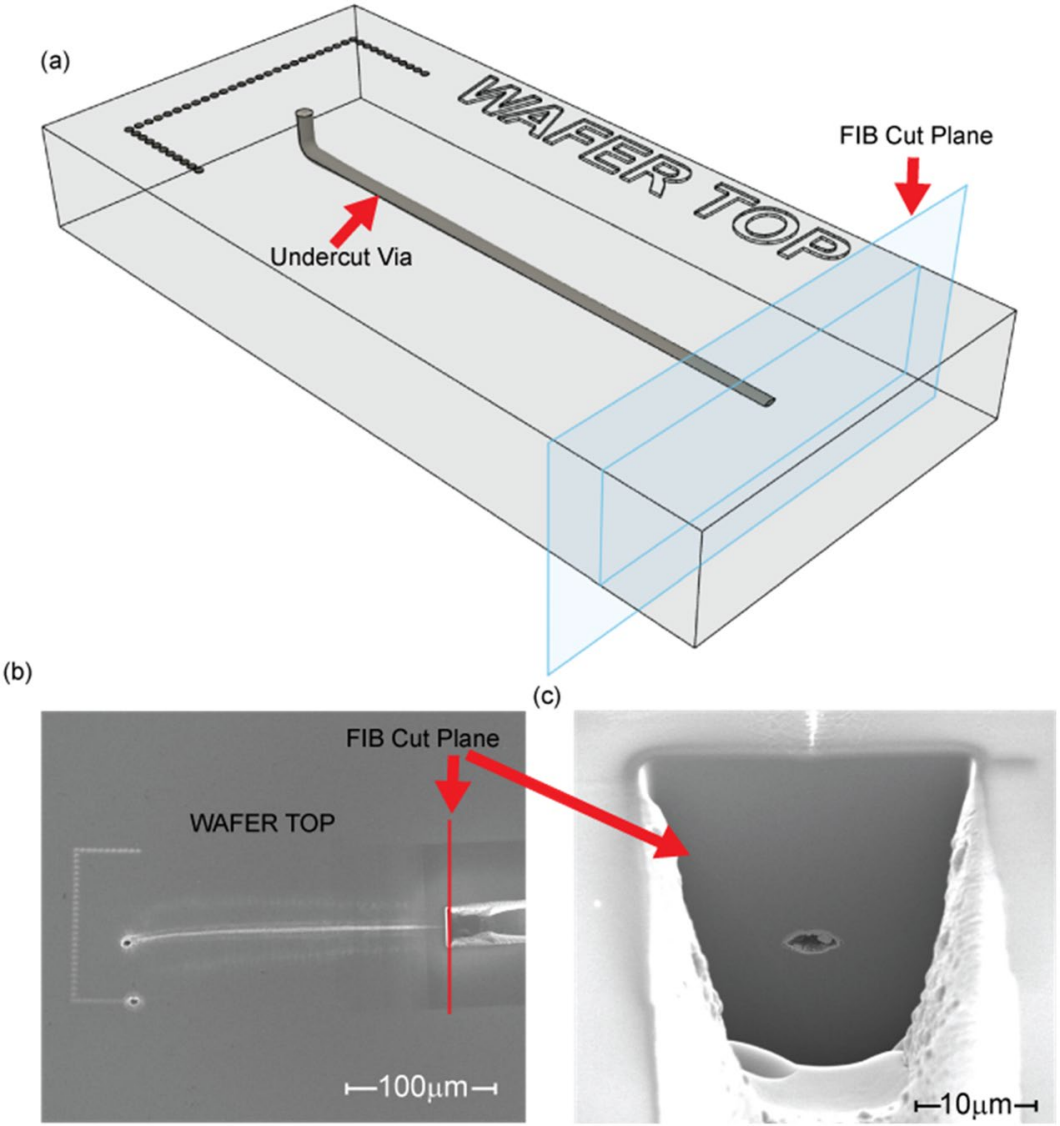
This via microchannel has an 18 µm long vertical section (i.e., perpendicular to the surface) followed by a 90° turn and a 232.5 µm long section that is horizontal (i.e., parallel) to the surface. (**a**) 3D illustration of the undercut via with the FIB cut plane labeled and the “C” shaped surface identification mark. (**b**) SEM image of the top-down view of the long undercut with the FIB cut labeled. (**c**) SEM image looking perpendicular to the FIB cut plane at the end of the undercut via revealing the end of the via microchannel 18 µm below the wafer surface.

Figure 4 shows a long undercut via etched in SiC. The via begins perpendicular to the surface and travels 18 µm down into the substrate. It then turns 90° and travels horizontally (parallel to the wafer surface) for 232.5 µm. Figure 4a shows a diagram of the via along with the plane where a focused ion beam (FIB) cross-section cut was performed to expose the end of the via. Figure 4b shows a SEM image of the top SiC surface. The “C” shaped marking on the surface is an aid in locating the via after the etch. There is also some top surface marking above the undercut via. Figure 4c shows the FIB cut plane view where the end of the via is exposed about 18 µm below the top surface. Since the only way this technique works is with access to the HF acid, we can conclude that the via is continuous.

**Figure 5:**
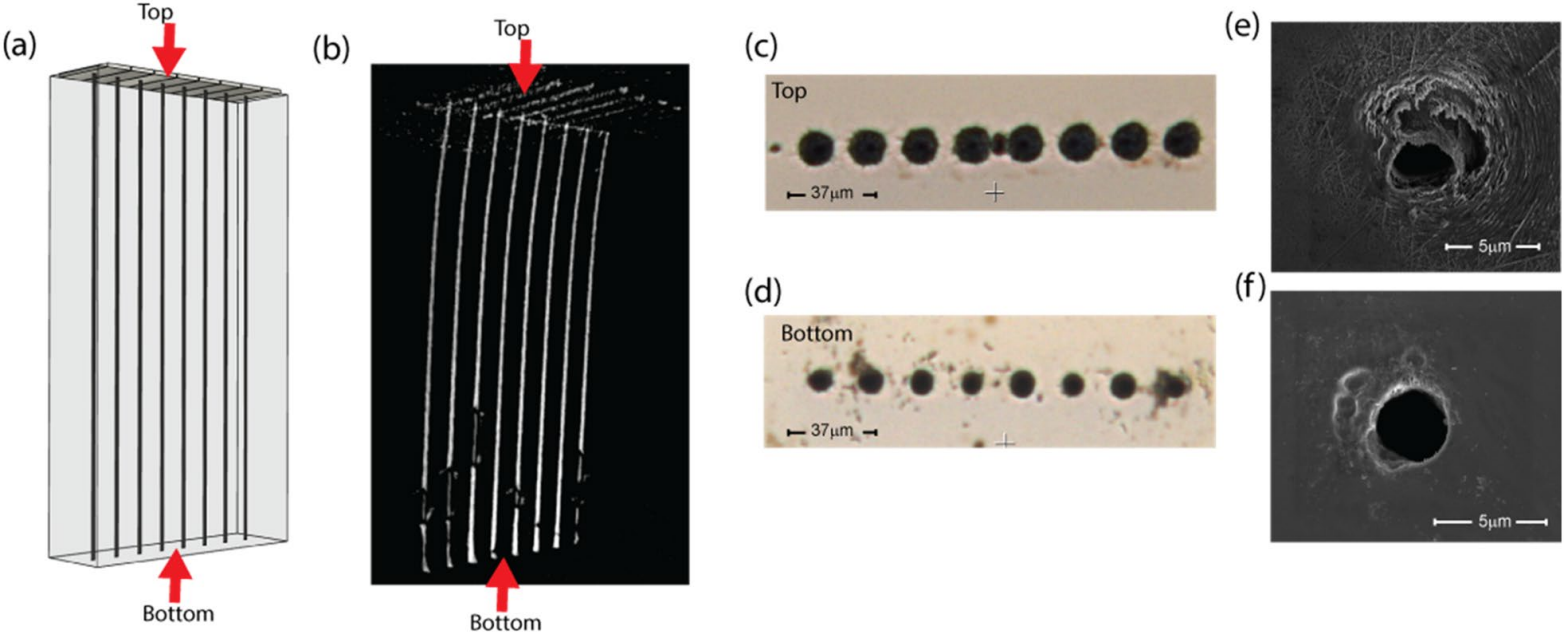
Eight high aspect ratio, 350 µm long through-wafer via microchannels in SiC with a center to center spacing of 22 µm. (**a**) 3D illustration of the 8 through wafer vias. (**b**) 3D image of the 8 through wafer vias using x-ray micro-computed tomography (x-ray micro-CT) reconstruction. (c,d) The top and bottom views of the 8 vias in an optical microscope. (e,f) The top and bottom SEM images of a via with similar characteristics to those in the 8 via example.

Figure 5 shows an image of eight vias etched in SiC that are spaced approximately 22 µm apart that go completely through the 350 µm thick SiC wafer. These eight vias were etched in parallel by splitting the beam using a holographic beam splitter. This etch was repeated in four different locations on the same wafer with the same result. Figure 5a is a 3D model showing an overview of the geometry of the eight vias with labeled top and bottom surfaces of the SiC. Figure 5b was taken non-invasively using a micro-computed tomography (micro-CT) x-ray imager with sub-micron resolution which allows for non-destructive imaging of subsurface features. A video and data of this 3D image are included in the “supplementary information”.

Figure 5c,d are optical microscope images of the vias entry holes from the top and bottom views respectively. Figure 5e,f show SEM images of the top and bottom view of a single through wafer via respectively, a via similar to those in the eight via etch. This via has a diameter of around 3.2 µm on both the top and bottom of the via. Since the via diameters observed in cross-section experiments are consistently either smaller than or the same size as the via entry and exit holes (except when intentionally made larger), this via is assumed to be 3.2 µm or less through the entire 350 µm thick wafer, giving an aspect ratio of at least 109:1.

## Discussion

The results presented in the previous section are good representations of the technique’s capabilities in creating non-line-of-sight, high aspect ratio vias in SiC. The cleave and polish process used to reveal the sub-surface features in Figs. 3 and 4 require the etches to remain in a single plane. However, this is not a limitation of this technique since any continuous, 3D structure can be made in bulk single-crystal SiC if the HF electrolyte is present at the etch surface. This capability not only makes direct connection interposers possible, but it creates the possibility for other features like microfluidic channels that are interleaved through the interposer for intra-chip cooling. The x-ray micro-CT imaging technique used in Fig. 5b is very attractive for imaging out-of-plane aspects of these 3D structures. Due to the lack of availability, no x-ray scans have been done on multi-plane 3D etches.

Unlike most other fabrication techniques, via diameter is not dependent on via length. For Figs. 3a–c and 4, this results in a consistent via diameter of 2–4 µm through the entirety of the etches. Figure 3c shows the smoothness and consistency of the via wall using this technique. Note that the periodic bumps on the via wall are due to the steps of the XYZ stage used for the demonstration (a higher quality stage would eliminate these features).

Direct write manufacturing techniques often suffer from slow fabrication times. Using this technique, we have achieved an etch rate of around 2 µm a minute regardless of via shape or complexity. No acid depletion effects were observed, but with longer structures the etch rate could drop further. At this rate, a via through a 350 µm wafer takes several hours. To increase fabrication speed, we have shown that parallel beams can be used. Figure 5 is an example of an experiment which used a 1 × 8 holographic beam splitter which effectively increases the etch rate by eight times. We have also proven this concept with a 4 × 4 holographic beam splitter.

Once an etched via has made it through the wafer, there is a very small amount of HF electrolyte that forms micro-bubbles on the other side. This HF evaporates quickly. The etch system is placed in a fume hood to ventilate these harmful HF fumes to avoid any damage to the optical setup.

## Conclusion

In summary, we have demonstrated the fabrication of non-line-of-sight via microchannels in SiC with aspect ratios up to at least 109:1, significantly higher than ever before achieved in SiC or other interposer materials. Additionally, we have demonstrated for the first time, three-dimensional via microchannels in single-crystal SiC that allow routing of vias within an interposer.

The novel fabrication technique that makes this possible combines two photon absorption with electrochemical etching in a manner where etching only occurs when both HF acid and positive charge carriers (“holes”) are present. Holes are created only in close proximity to the laser focus by using a femtosecond laser in combination with a high NA focusing objective, resulting in very high etch selectivity between the SiC in the focal spot and the SiC outside the focal spot. The result is a wet etching process that can create vias with a diameter of around 3 µm and an arbitrary length. The arbitrary length enables the vias to have a very high aspect ratio. In this work, we used this technique to fabricate vias with a length up to 350 µm.

The SiC wafer is transparent to the 515 nm femtosecond laser light, which has a photon energy below the bandgap energy of SiC. Thus, the laser focus can be placed at any location within the SiC wafer. If the SiC at or near the location of the focus is also in contact with HF acid, etching occurs. Thus, the via fabrication does not require line-of-sight access. We have taken advantage of the non-line-of-sight feature of this technique to create both non-direct through wafer vias and horizontal vias (i.e., microchannels that run parallel and underneath the surface of the SiC wafer).

With this technique, we have created 3-dimensional microstructures in SiC with higher aspect ratios than ever before demonstrated and via microchannels in SiC that provide flexibility in subsurface routing for the first time. This novel technique has the potential to enable IC manufacturers to increase the spatial efficiency of interposers, reduce the length of interconnects, provide higher interconnect densities between chiplets, and remove heat more efficiently than what is possible with current interposers used today for 3D integration.

We have demonstrated parallelized fabrication using a 1 × 8 holographic beam splitter. With a higher-powered laser, we suspect that 1000 parallel beams or more could be used to further increase the practicality of this technique.

Further research is required to develop a consistent technique for these super high-aspect ratio vias with a low resistance conductor; however, most high-aspect ratio vias are filled using an electrodeposition technique^35–37^. This is likely the technique that we will use to fill these 3D vias.

## Methods

### Experimental setup

**Figure 6:**
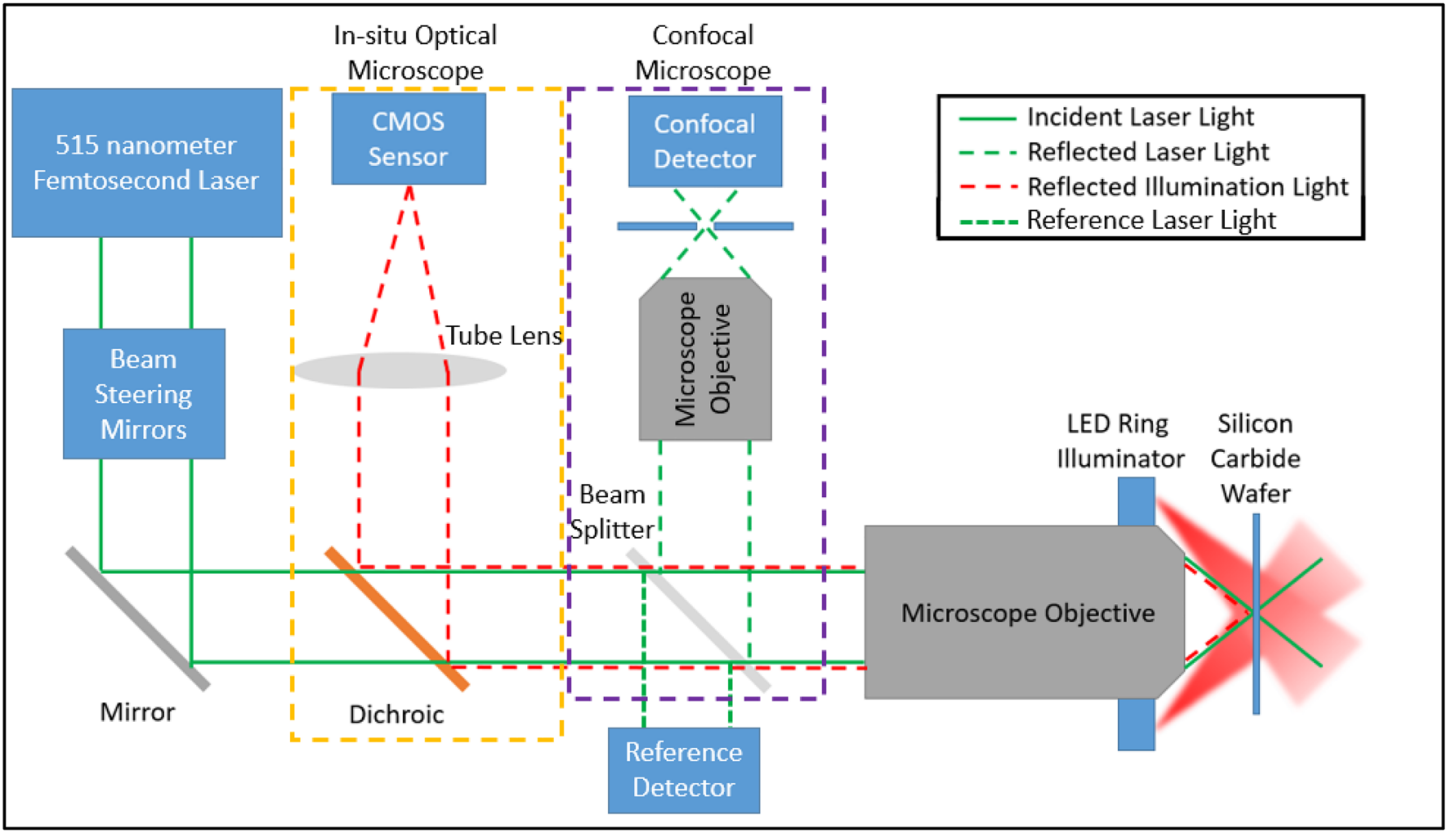
Optical system for two-photon photoelectrochemical etching process.

The main purpose of the experimental setup is to generate as many holes as possible through two-photon absorption at the laser focus and no holes at any other locations. Since the amount of two-photon absorption is proportional to the intensity of the light squared, the main goal is to get as intense of a focus as possible at the etch surface and have the intensity fall off with distance away from the focus. Figure 6 shows the experimental system that is designed to create the high intensity focus. The two important pieces for successful etching are the femtosecond laser and the high numerical aperture (NA) microscope objective. The laser is an Amplitude System’s Satsuma femtosecond laser with a harmonic doubler to produce the 515 nm light. The femtosecond laser compresses its output power into a small pulse with a total energy of 9 µJ and pulse width of 290 fs. The laser has a repetition rate of 250 kHz resulting in a peak pulse power of about 35 MW with an average power of 2.5 W. With this low average power, no localized heating was observed. The microscope objective is a Mitutoyo Plan Apo 100 × objective, which has a numerical aperture of NA = 0.7 resulting in minimum spot with a full width at half maximum of FWHM = 478 nm. With these two components, the system creates a very high intensity spot at the etch surface which creates a large number of electron–hole pairs to assist in the chemical reaction.

Figure 6 shows that the laser beam is directed by a series of mirrors through a beam splitter that reflects 0.6% of the beam which is collected by a reference detector. The laser power is monitored and controlled using the reference beam. The power at the focal spot is maintained at a level below the ablation threshold for the SiC but at a high enough level to achieve two-photon absorption. The main beam passes through the beam splitter to the high NA, long working distance microscope objective. This objective focuses the light to a submicron spot on the surface of the SiC, where some of the light is absorbed due to two-photon absorption, some of the light is transmitted, and some of the light is reflected back through the objective lens. The reflected light is directed into the confocal microscope objective which focuses the reflected light through a pinhole and into a photodetector. The pinhole is aligned such that the maximum power in the confocal detector occurs when the focus of the main objective is directly focused on the surface of the SiC wafer. This effectively creates a surface detector. In addition, there is an optical microscope. The microscope uses the main objective lens as the objective for the microscope. A ring light is placed either around the objective or behind the SiC wafer to provide light to the surface of the wafer where the objective lens is focused. The light from the ring is reflected or transmitted respectively through the objective and is directed by a dichroic mirror through a tube lens and into a CMOS camera. The dichroic mirror is meant to transmit most of the laser light and reflect most of the illumination light. This optical microscope system allows us to inspect the surface of the wafer before and after etching. The whole system is placed on an isolation stage to reduce vibrations in the system and in a fume hood to ventilate harmful fumes.

**Figure 7:**
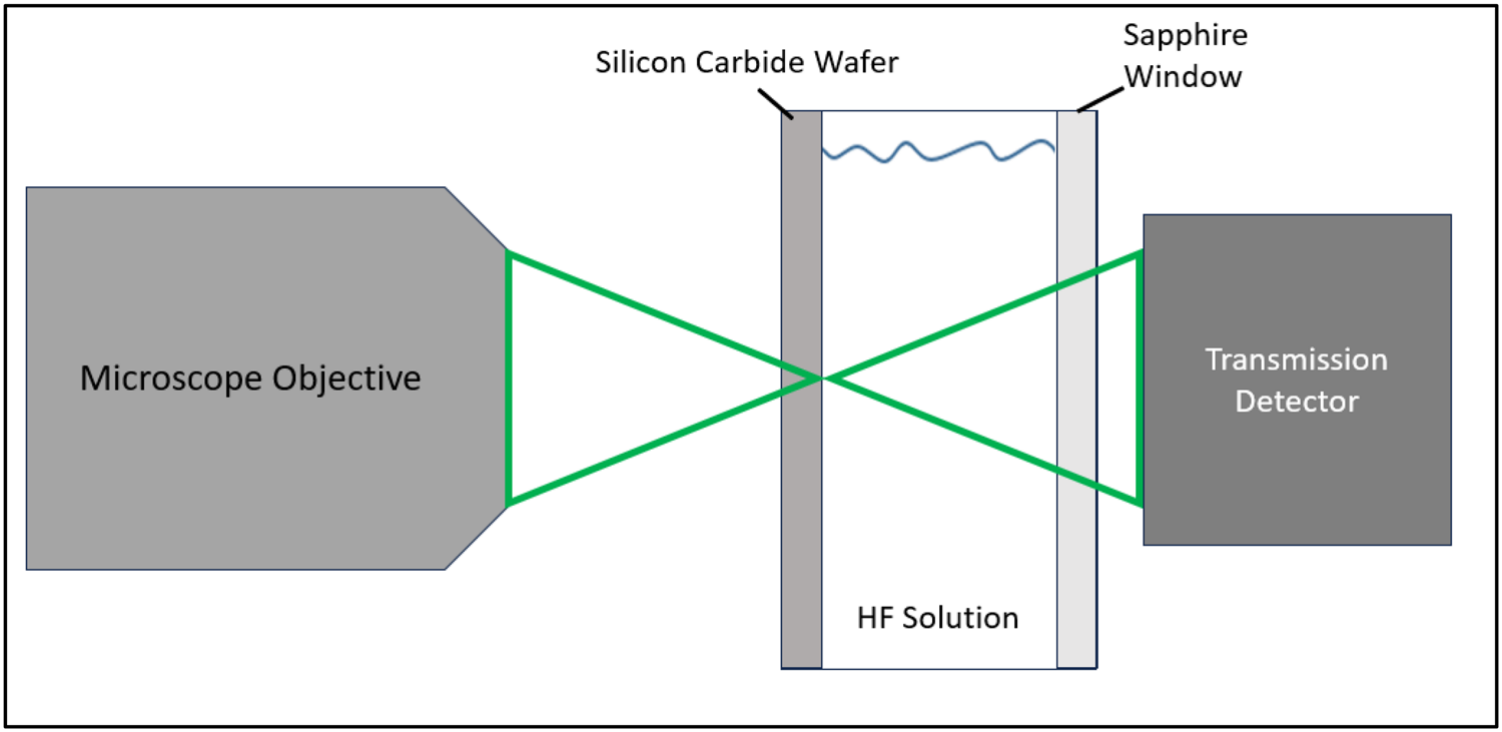
Diagram showing the main objective focusing on the back side of the SiC wafer.

Figure 7 shows a closer view of the focus of the main objective in the system. The light coming through the objective is focused on the back surface of the SiC. In these demonstrations, we used n-type single-crystal 4H–SiC for our substrate. A reservoir of hydrofluoric acid (HF) solution (5% HF, 10% ethanol, 85% deionized water) is in contact with the back surface so that it is available at the etching sight. Etches are started from the backside of the wafer to avoid any scattering that would occur if the laser light passed through a previously etched portion of the wafer before reaching the etching surface. The other wall of the reservoir is a sapphire window since it is chemically resistant to HF and it is transparent to the 515 nm laser wavelength. A transmission detector is placed behind the chamber to monitor drops in transmitted power due to two-photon absorption.

The chamber is made from high-density polyethylene (HDPE) which is resistant to the HF solution. The SiC wafer and Sapphire window are set between Viton O-rings and the chamber is screwed together to create a watertight seal. The chamber is then bolted down to an XYZ translation stage to help reduce vibrations. During the etch, the translation stage moves the etching chamber relative to the fixed laser focus. This effectively moves the focus to different locations on the wafer, allowing for precisely controlled etch locations.

Due to variability in the laser absorption of different 4H-SiC wafers, the power used to etch varies significantly from etch to etch. There is also a significant difference in two photon absorption at the back surface relative to the front surface. This is probably caused by a drop in intensity due to slight linear absorption (caused by doping) and beam axial beam spread from spherical aberrations (since SiC has a high refractive index). Therefore, to have consistent etch parameters throughout the thickness of the wafer, the laser power must be dynamically varied throughout the etch dependent on depth. Typical laser powers range from 0.1–2 mW (this is average laser power not peak power) at the input of the objective. At these powers, we saw an etch rate of about 2 µm per minute.

### Imaging techniques

Verification of the technique requires determining what is happening under the surface. If a through wafer via is etched in SiC, there is only a small hole on the front and back surface that are visible. When focused only on those surfaces, there is no indication of what is going on in the bulk of the material. Imaging the structures is accomplished in several ways. The techniques used in this work include focused ion beam (FIB) cutting with scanning electron microscope (SEM) imaging, ablation cleaving with polishing and SEM imaging, and x-ray micro-computed tomography (x-ray micro-CT).

FIB cutting is a very precise and easy way to look at features that are less than 30 µm below the surface. It works by directing a focused beam of ions at the surface of the material. The ions have enough energy to remove material from the SiC surface. This technique is used in Fig. 4 to reveal the end of the etch. This tool is used in conjunction with SEM imaging that makes it very precise and convenient to image after the cut has been made; however, FIB cuts suffer from the redeposition of materials, so the observed depth is limited. It also destroys the structure of interest. The image brightness of the SEM images were modified using PowerPoint.

**Figure 8:**
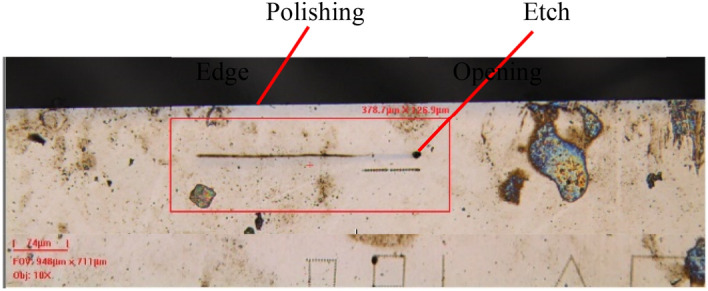
Optical microscope image of the etch in Fig. 3 near an edge that was cleaved using our ablation assisted cleaving technique. The edge was polished after cleaving when this image was taken.

The next technique uses laser ablation-cleave and diamond polishing. This uses the same laser system to damage (ablate) the SiC very close to the desired etch. If the via microchannel is a non-line-of-sight via, then the ablation line is parallel to the via. The wafer is then cleaved by pressing either side of the wafer over a fulcrum. The wafer piece is then polished down to the via microchannel using an edge polisher fitted with diamond lapping plates. The wafer edge is polished until half of the etch is polished away. This is difficult since the etches can be less than 3 µm in diameter. Figure 8 shows the wafer piece that was ablated and cleaved prior to polishing into the long undercut etch shown in Fig. 3b of the paper. This is an effective way to image entire vias if the via lie in a single plane. However, it is a destructive technique and destroys the etch that is being imaged.

The next technique uses a Zeiss Xradia Versa 620, which is a 3D x-ray microscope. This equipment utilizes a series of x-rays images to see inside an object using micro-computed tomography (micro-CT) with resolution down to about 600 nm. The substrate is illuminated with an x-ray beam, and sensors on the other side of the substrate measure the portion of the beam that is not absorbed by the substrate. In the case of the vias, small gaps of air or metal (if the via microchannels are filled) cause small differences in absorption on the other side. Measurements are taken at different angles. The various 2D images are stitched together using image reconstruction techniques to create a 3D image. Figure 5b of the paper is a snapshot of a 3D image or model created using this technique. A video of the 3D model is included in the “supplementary information”. These images were reconstructed using the ImageJ image processing software. This tool is a good way to image structures in SiC without destroying the actual structure, but to attain a high enough resolution, the piece being imaged needs to be small (approximately one centimeter or less) which makes it difficult to characterize the geometry of structures while the wafer is still intact.

### Supplementary Information

Supplementary Video 1.

Supplementary Information 1.

## Data Availability

All data sets will be made available upon reasonable request to the corresponding author (paynejar@byu.edu).
