# Supplementary material for: Fabrication of high aspect ratio, non-line-of-sight vias in silicon carbide by a two-photon absorption method
Source: Sci Rep. 2024 Jan 25;14:2176. doi: 10.1038/s41598-024-52672-6 (PMC10810901; doi:10.1038/s41598-024-52672-6)
Supplement: Supplementary file 2 — Supplementary Information 1. [file 41598_2024_52672_MOESM2_ESM.doc]

Rotating Computed Tomography 3D Scan of 8 consecutive vias:

This video gives further context to the computed tomography image shown in Figure 5b of the paper. The vias travel all the way through the 350 µm thick wafer. They are about 6 µm in diameter and about 22 µm apart. The 3D image was reconstructed from raw x-ray data scans using ImageJ image processing software.
